# Cuprizone Intoxication Results in Myelin Vacuole Formation

**DOI:** 10.3389/fncel.2022.709596

**Published:** 2022-02-18

**Authors:** Sarah Joost, Felix Schweiger, Friederike Pfeiffer, Carolin Ertl, Jonas Keiler, Marcus Frank, Markus Kipp

**Affiliations:** ^1^Institute of Anatomy, Rostock University Medical Center, Rostock, Germany; ^2^Werner Reichardt Centre for Integrative Neuroscience, University of Tübingen, Tübingen, Germany; ^3^Medical Biology and Electron Microscopy Center, Rostock University Medical Center, Rostock, Germany; ^4^Department of Life, Light and Matter, University of Rostock, Rostock, Germany; ^5^Center for Transdisciplinary Neurosciences Rostock, Rostock University Medical Center, Rostock, Germany

**Keywords:** multiple sclerosis, oligodendrocyte injury, myelin pathology, myelin-axon interaction, myelin ultrastructure, serial block face scanning electron microscopy, vacuoles

## Abstract

Myelin damage is a histopathological hallmark of multiple sclerosis lesions. Results of *post mortem* studies suggest that impaired myelin-axon interaction characterized by focal myelin detachments is an early event during lesion genesis. In this study, we investigated the ultrastructural changes of the axon-myelin interface in the cuprizone model using serial block face scanning electron microscopy and immunohistochemistry. We show that non-inflammatory injury of oligodendrocytes by cuprizone intoxication results in myelin vacuole formation and axonal swellings, paralleled by early alterations of the node of Ranvier cytoarchitecture. This remarkable resemblance of ultrastructural myelin characteristics in multiple sclerosis and the cuprizone animal model suggests that the cuprizone model is a valuable tool to study early pathologies during lesion formation.

## Introduction

Multiple sclerosis (MS) is an inflammatory demyelinating disease of the central nervous system (CNS), leading to irreversible neuronal damage and, in consequence, clinical disability. Different clinical disease courses are known. Most patients initially present with a relapsing-remitting disease course (RRMS) which is, on the clinical level, characterized by recurrent episodes of new or worsening symptoms (relapses) and periods of stability in between those relapses. It is believed that focal lesions of inflammatory demyelination result in the clinical deficits observed during the RRMS disease stage. On the histopathological level, such lesions are characterized by peripheral immune cell recruitment, destruction of oligodendrocytes and the myelin sheaths, glia cell activation (microglia and astrocytes) as well as acute axonal injury (Kipp et al., 2012).

The histopathological hallmark which distinguishes MS from other CNS disorders is the formation of large confluent plaques of primary demyelination. For this reason, any concept of MS pathogenesis has to provide an explanation for this highly specific destruction of the oligodendrocyte-myelin-axonal unit. The sequence of molecular events leading to oligodendrocyte loss and consequently demyelination are not fully understood. Different stressors are known which can induce oligodendrocyte degeneration including oxidative stress, mitochondrial dysfunction, nitric oxide, protein misfolding or inflammatory cytokine exposure (Smith and Lassmann, 2002; Aboul-Enein and Lassmann, 2005). Of note, viable oligodendrocytes and an intact myelin sheath are indispensable for neuronal health. Oligodendrocytes provide nutritional support to neurons (Funfschilling et al., 2012), fast axonal transport depends on oligodendrocytes (Edgar et al., 2004), and mice deficient for mature myelin proteins display severe neurodegeneration (Uschkureit et al., 2000).

To gain insight into the process of early demyelination, several studies on the ultrastructure of the myelin-axonal unit during demyelination have been conducted. Romanelli et al. (2016) found bulb-like myelin detachments (called myelinosomes) both in inflammatory lesions of different mouse models of experimental autoimmune encephalomyelitis (EAE), a common MS model, and in active lesions of post mortem MS patient material. Comparably, Weil et al. (2016) reported swellings of the inner tongue of myelin in EAE, an experimental focal Neuromyelitis optica model and the cuprizone model. In a recent study, Luchicchi et al. (2021) observed myelin detachments from axons (called blisters) in the non-inflamed, normal-appearing white matter (NAWM) of MS patient tissue, paralleled by alterations of the node of Ranvier cytoarchitecture and an increase in citrullinated myelin proteins.

We and others recently demonstrated that metabolic oligodendrocyte stress, induced by intoxication with the mitochondrial toxin cuprizone, results in myelin basic protein (MBP) citrullination which triggers autoimmune inflammatory demyelination (Scheld et al., 2016; Caprariello et al., 2018; Kaddatz et al., 2021). The ultrastructure of the myelin-axonal unit in the cuprizone model has been described in early works, demonstrating swellings of the inner tongue of the myelin sheath similar to the observed myelin detachments in MS and the EAE model (Blakemore, 1973; Ludwin and Johnson, 1981). In this study, we aimed to deepen the understanding of the myelin ultrastructure during demyelination in the cuprizone model. For this purpose, we took advantage of serial block face scanning electron microscopy (SBF-SEM) (Denk and Horstmann, 2004; Jurrus et al., 2009) to investigate pathological alterations at the axonal-myelin interface in the cuprizone model.

## Materials and Methods

### Animals and Region of Interest

Eight-week-old C57BL/6 male mice (*N* = 20) were used and the experiments were approved by the government of Upper bavaria/Regierung von Oberbayern (#55.2-154-2532-73-15). The experimental procedures were performed according to the Federation-of-European-Laboratory-Animal-Science-Associations recommendations. Cuprizone intoxication (0.25%) was induced as described previously (Hochstrasser et al., 2017). Since cuprizone-induced demyelination follows a region and time dependant pattern, all analyses were performed in the midline of the corpus callosum at the level of the rostral hippocampus (∼plate 64 according to the coronal Allen Mouse Brain Atlas).

### Ultrastructural Analysis via Serial Block-Face Scanning Electron Microscopy and Transmission Electron Microscopy and Quantifications

Myelinated and demyelinated corpora callosa (*N* = 4 each) of 3 weeks cuprizone-intoxicated mice were processed for SBF-SEM as published (Ohno et al., 2014). In brief, imaging was performed by using a Sigma VP scanning electron microscope (Carl Zeiss) equipped with a 3View in-chamber ultramicrotome system (Gatan). Images were processed and measured with the open source program Reconstruct (BU, Boston, MA, United States) or Imaris 8.4 (Bitplane, United States). Additionally, corpora callosa of 1 and 3 weeks cuprizone-intoxicated mice (*N* = 3 each) were processed for toluidine blue stains and transmission electron microscopy (TEM) as published previously (Ebner et al., 2018).

To quantify the relative frequency of myelin detachments in control and cuprizone-intoxicated mice, a virtual counting frame (area 4757 μm^2^) was randomly placed within the first image of the *z*-stack. For reducing any bias, the 100th image (correlating with 8 μm in depth) of the *z*-stack was chosen as a starting point. In order to increase the number of evaluated axons images no. 300 (correlating with 24 μm depth) and no. 500 (correlating with 40 μm depth) were included in the analysis. A myelin detachment was counted only if an axon was clearly visible within the vacuoles.

To assess whether small- or big-caliber axons are preferentially vulnerable to develop myelin detachments, in a first step we quantified axonal diameters (without the surrounding myelin sheath) in control mice. To this end, four virtual counting frames (areas 125 μm^2^ each) were randomly placed within the center of the *z*-stack, and the diameters of axons within the virtual counting frames were measured at six different *z*-stack positions. For each single axon, the results were averaged. In a next step, 15–25 virtual counting frames (areas 334 μm^2^ each) were randomly placed within the first third of the *z*-stack of cuprizone-intoxicated mice, and the 50 adjacent images (in both directions of the *z*-stack) were investigated for the presence of a myelin detachment. In cases where myelin detachments were detected within the virtual counting frames, respective axonal diameters were measured at six different *z*-stack positions distant to the myelin detachments. Again, for each single axon, the results were averaged.

To quantify the relative frequency of partial versus complete myelin detachments, the myelin detachments analyzed above for axonal diameter measurements were followed in the *z*-plane and their morphology was assessed.

### Histological Analyses

All histological analyses were performed with paraffin-embedded brain sections. To label the myelin protein myelin proteolipid protein (PLP), nodal ankyrin-G or paranodal contactin-associated protein (Caspr), the following primary antibodies were used: mouse-anti-ankyrin G (Antibodies Incorporated Cat# 73-146, RRID:AB_10697718, 1:750), mouse-anti-PLP (Bio-Rad Cat# MCA839G, RRID:AB_2237198, 1:5000) and rabbit-anti-Caspr (Abcam Cat# ab34151, RRID:AB_869934, 1:3000). Appropriate negative controls (omission of primary antibody) were performed in parallel. ImageJ (version 1.52p) was used to evaluate the relative staining intensities using semi-automated densitometrical evaluation after automatic threshold setting (threshold Moments for ankyrin G, Rényi entropy for Caspr) and to perform automated counting of Caspr^+^ or ankyrin G^+^ particles (particle size min. 4 pixel) after threshold settings were manually adjusted in a blinded manner to obtain the best signal to noise ratio (based on the threshold Rényi Entropy) (Schneider et al., 2012). Please note that automated ankyrin G^+^ particle density analysis was of limited efficiency in control sections because ankyrin G^+^ spheroids often were in close proximity to each other due to their high density and could not always be discriminated by the algorithm. For Caspr analysis, optical density measurement was inferior to particle recognition because in 3 weeks cuprizone-intoxicated mice, areas of blurry Caspr-immunoreactivity emerged, possibly due to acute paranodal disintegration. However, we decided to provide both analyses for optimal comparability of ankyrin G and Caspr stains.

### Electrophysiology

**FIGURE 1:**
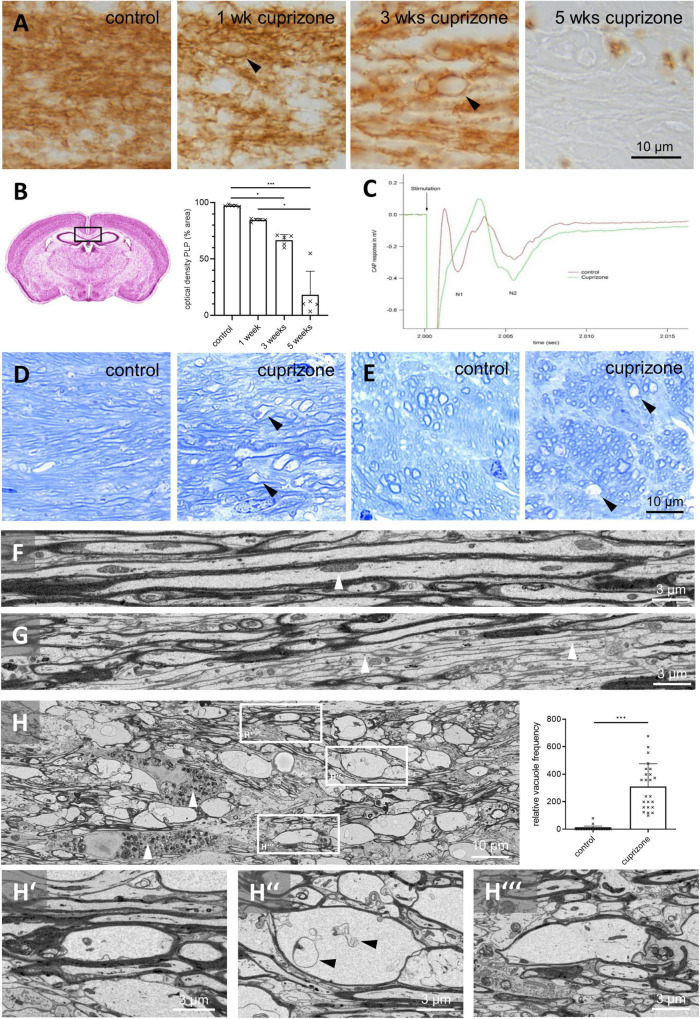
Vacuolization of myelin presents more frequently in cuprizone-intoxicated than in control mice. **(A)** Anti-PLP stains of the midline of the corpus callosum in control and cuprizone-intoxicated mice. Black arrowheads indicate vacuoles. **(B)** Optical density of PLP during cuprizone intoxication in the medial corpus callosum. Statistical testing by Kruskal–Wallis test (*p* = 0.0005) followed by Dunn’s multiple comparisons test, *n* = 5 mice per group, Ctrl – 3 weeks: *p* = 0.0453, Ctrl – 5 weeks: *p* = 0.0004, 1 week – 5 weeks: *p* = 0.0452. **(C)** Representative result of compound action potential measurements in control (green) and 3 weeks cuprizone-intoxicated (red) mice. Each trace is averaged from 18 to 20 single measurements from the same tissue slice. Note that the N1 peak amplitude decreased in cuprizone-intoxicated mice compared to controls. Further note that for the unmyelinated axons, an increase in amplitude was seen in the N2 peak amplitude. **(D,E)** Toluidine blue-stained sections of control and 3 weeks cuprizone-intoxicated mice [corpus callosum **(D)** and cingulum **(E)**]. Note the vacuolated appearance (black arrowheads) in both white matter tracts in cuprizone-intoxicated mice. **(F,G)** Representative SBF-SEM images demonstrating myelinated **(F)** and non-myelinated [**(G)**, white arrowheads] axons in control mice. The white arrowhead in **(F)** highlights an intra-axonal mitochondrion. **(H)** Representative SBF-SEM image demonstrating the appearance of vacuoles in white matter tracts after 3 weeks of cuprizone intoxication [white boxes, shown at higher magnification in **(H’–H”’)**] and relative frequency of vacuoles in control and 3 weeks cuprizone-intoxicated mice, evaluated in SBF-SEM image *z*-stacks. Statistical testing by Mann–Whitney test, *N* = 4 mice, *n* = 6 *z*-stacks per mouse, *p* < 0.0001. White arrowheads in **(H)** highlight axonal myelin-laden microglia. *p ≤ 0.05 and ^***^p ≤ 0.001.

Compound action potentials (CAPs) across the corpus callosum were recorded in three animals per group as previously described (Pfeiffer et al., 2019). In brief, after anesthesia induction and decapitation of mice, 400 μm thick coronal brain slices were prepared using a vibratome, transferred to an incubation chamber and gradually cooled down to room temperature, allowing the slices to equilibrate in Ringer solution. Measurements were then performed using a recording chamber mounted on the stage of an upright microscope (FN-1, Nikon, Japan). The recording and the stimulation pipette were placed at the same distance from the midline in fiber bundles of the caudal part of the corpus callosum, at a total distance of 1–1.4 mm distance from each other. Per sweep, one pair of pulses (with an interpulse interval of 40 ms) was applied every 10 s, each pulse with a duration of 100–250 μs with an isolated pulse stimulator (A-M Systems, Model 2100, Science Products, Germany). 18–20 sweeps per animal were averaged into a single trace as representatively shown in Figure 1C.

### Statistical Analyses

All data are given as the arithmetic means ± SEM. Differences between groups were statistically tested using the software package GraphPad Prism 5 (GraphPad Software Inc., San Diego, CA, United States). All data sets were tested for normal distribution by Kolmogorov–Smirnov test (significance level alpha = 0.05). Further testing for significant differences between groups was conducted as stated in the figure legends with confidence intervals of 95%. The following symbols are used to indicate the level of significance: *p ≤ 0.05, ^**^p ≤ 0.01, ^***^p ≤ 0.001.

## Results

To induce demyelination, mice were intoxicated with cuprizone for up to 5 weeks. Demyelination was visualized by immunohistochemical staining of the myelin protein PLP. In the medial corpus callosum, PLP immunoreactivity decreased over the course of cuprizone treatment indicating progressive demyelination (Figures 1A,B). To investigate whether demyelination was paralleled by functional deficits, electrically evoked CAPs were recorded across the corpus callosum in control and 3 weeks cuprizone-intoxicated mice. Typically, CAPs consist of two or more downward deflections referred to as N1 and N2. The first downward deflection, N1, represents the faster, myelinated axons, while unmyelinated, or partially myelinated axons contribute to the second downward deflection, N2. Compared to controls, we observed an almost complete loss in the myelinated component of the N1 peak amplitude after 3 weeks cuprizone, paralleled by an increased N2 peak amplitude (Figure 1C) hinting at severe functional myelin damage with myelin protein abundance at a decreased level (Figure 1B).

On close inspection of the anti-PLP stained sections, vacuole-like structures were evident especially after 3 weeks of cuprizone intoxication, and occasionally also found after 1 week of cuprizone intoxication (arrowheads in Figure 1A). To confirm this observation, we analyzed semi-thin sections of mice after 3 weeks of cuprizone by toluidine blue staining. In semi-thin sections of control mice, longitudinally cut axons with different diameters were clearly visible, and the myelin sheaths were closely attached to the axonal surfaces (i.e., axolemma; Figure 1D). The same morphology was observed in the cingulum, where axons were cut in the transversal plane (Figure 1E). In cuprizone-intoxicated mice, both regions had a vacuolated appearance with several irregular, ovoid-shaped, non-stained areas (see arrowheads in Figures 1D,E).

To understand the ultrastructure of such vacuoles, we used SBF-SEM and, in a separate experiment, TEM. A typical corpus callosum section showed a few larger myelinated axons (Figure 1F) interspersed among more numerous small myelinated and unmyelinated axons (arrowheads in Figure 1G). Within the axons, we frequently observed intraaxonal organelles, like mitochondria (arrowhead in Figure 1F). After 3 weeks of cuprizone intoxication, the most prominent pathology was the presence of numerous electron pale, ovoid structures which were surrounded by an electron dense sheath, resembling the observed vacuoles (white boxes in Figure 1H, high power views in Figures 1H’–H”’ and Supplementary Videos 1, 2). Vacuoles were not observed in direct proximity to nodes or paranodes but situated within the internodes (Supplementary Figure 1). Frequently, these vacuoles contained irregular shaped membrane-like structures (arrowheads in Figure 1H”). Unbiased quantifications (see section “Materials and Methods”) revealed virtually no vacuoles in control mice (mean 4.9 ± SEM 3.6 counts/virtual counting frame), whereas numerous were found in the corpus callosum of cuprizone-intoxicated mice (310.1 ± 33.8 counts/virtual counting frame, Figure 1H).

**FIGURE 2:**
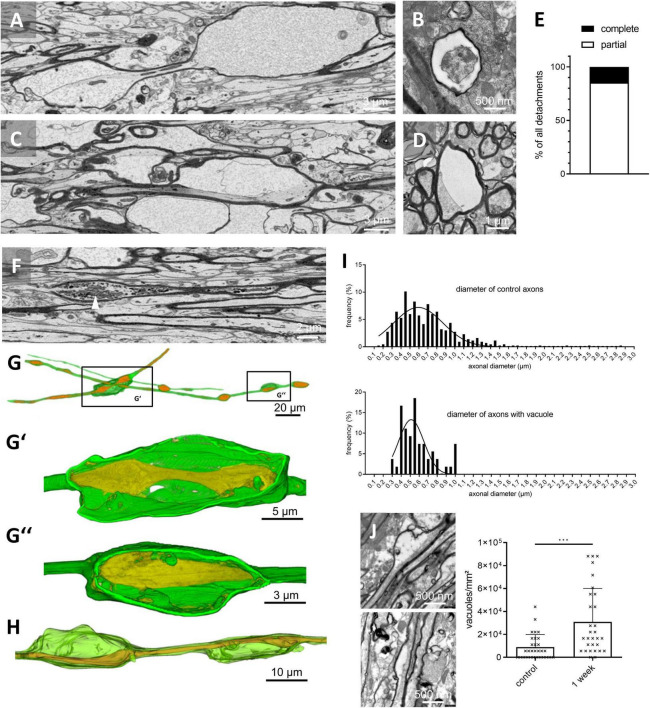
Fine structure of myelin vacuolization. **(A)** Representative SBF-SEM image of a vacuole with detachment of the entire myelin sheath from the axonal surface. **(B)** Representative TEM image of a vacuole with detachment of the entire myelin sheath from the axonal surface; transversal section. **(C)** Representative SBF-SEM image of a vacuole with partial detachment of the myelin sheath from the axonal surface. **(D)** Representative TEM image of a vacuole with partial detachment of the myelin sheath from the axonal surface; transversal section. **(E)** Relative frequency of complete versus partial detachments after 3 weeks cuprizone intoxication evaluated in 55 randomly chosen vacuoles in SBF-SEM image *z*-stacks, *N* = 3 mice, *n* = 15–25 vacuoles per mouse, 55 in total. **(F)** Representative SBF-SEM image of an axonal swelling (i.e., bleb). The white arrowhead highlights intra-axonal organelles. **(G,H)** Three-dimensional reconstructions of different vacuoles. **(G’)** Reconstruction of a vacuole with detachment of the entire myelin sheath from the axon surface. Note the axonal swellings within the vacuole. **(G”)** Reconstruction of a vacuole with partial detachment of the myelin sheath from the axon. **(H)** Reconstruction of the vacuole shown in **(C)**. **(I)** Frequency of axonal diameters in control mice (upper histogram, *N* = 3 mice, *n* = 435 axons) and of axonal diameters of axons affected by myelin detachment after 3 weeks of cuprizone intoxication (lower histogram, *N* = 3 mice, *n* = 54). **(J)** Representative TEM images of different vacuoles at week 1 and quantification of the vacuole density in control and 1 week cuprizone intoxicated mice. Statistical testing by Mann–Whitney test, *N* = 3 mice, *n* = 10 images per mouse, *p* = 0.0002. ^***^p ≤ 0.001.

These vacuoles resulted either from the detachment of the entire myelin sheath from the axon surface (i.e., the entire circumference of the myelin sheath was found to be detached from the axolemma; Figures 2A,B and Supplementary Videos 1, 2) or a partial detachment, where parts of the myelin sheath were still continuous with the axolemma (Figures 2C,D and Supplementary Videos 3, 4). To quantify the relative frequencies of both, partial versus complete detachments, 55 detachments were randomly selected and the relative proportions were quantified. As demonstrated in Figure 2E, ∼85% of the detachments were partial, whereas ∼15% were of the complete type. Furthermore, we found axonal enlargements surrounded by a morphologically intact myelin sheath (i.e., blebs; arrowhead in Figure 2F and Supplementary Videos 3, 4) and vacuoles with degeneration of the entire axon (not shown). 3D-reconstructions revealed (i) occasional axonal swellings within the vacuoles and (ii) that individual axons might bear several vacuoles (Figures 2G,H).

Next, we were interested whether small- or big-caliber axons are preferentially vulnerable to develop myelin detachments. Therefore, we first measured axonal diameters in randomly selected axons of control mice. In line with previous results (Sturrock, 1980; Sepehrband et al., 2016) and as shown in Figure 2I (upper histogram), axonal diameters in control animals ranged from 0.1 μm to 2.9 μm with high frequencies around 0.4–0.8 μm. Next, we repeated the analysis in cuprizone-intoxicated mice, just including axons showing myelin detachments. We found a shift of the frequency of the affected axons toward smaller axonal diameters (Figure 2I, lower diagram, a significant difference was found using Kolmogorov–Smirnov testing to compare cumulative distributions, *p* = 0.0121), suggesting that not big-caliber but small-caliber axons are preferentially affected by the myelin detachments in this model.

To investigate, whether vacuoles can as well be found after 1 week cuprizone intoxication, before demyelination is evident, additional TEM-experiments were performed. As demonstrated in Figure 2J, vacuoles were as well observed at week 1, albeit at a lower frequency (0.9 × 10^4^ ± 11.1 × 10^4^ vacuoles/mm^2^ in control; 3.1 × 10^4^ ± 2.9 × 10^4^ vacuoles/mm^2^ in cuprizone), confirming the observations from anti-PLP immunohistochemical stainings (Figure 1A).

**FIGURE 3:**
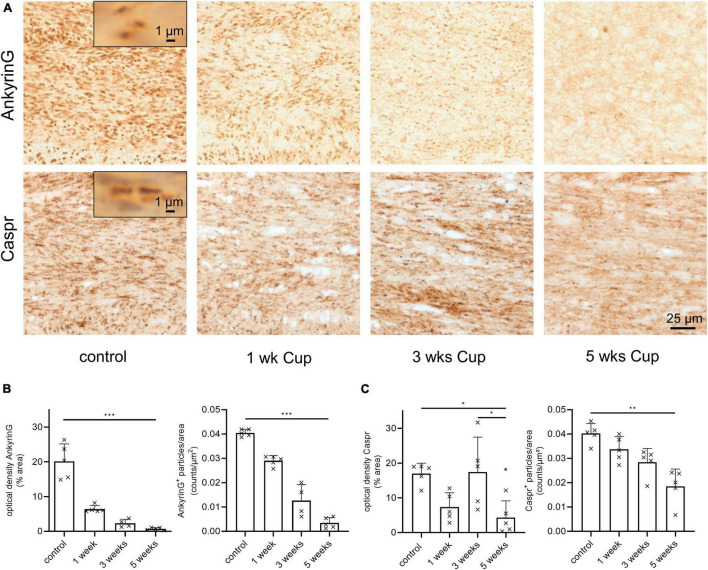
(Para)nodal pathology in cuprizone-intoxicated mice. **(A)** Representative anti-ankyrin G (upper row) and anti-Caspr stains during the course of cuprizone-induced demyelination. **(B)** Unbiased quantification of anti-ankyrin G staining intensities by optical density and particle density. Statistical testing by Kruskal–Wallis test followed by Dunn’s multiple comparisons test, *n* = 4–5 mice per group, optical density: Kruskal–Wallis test: *p* = 0.0007, Dunn’s multiple comparisons test: Ctrl – 5 weeks: *p* = 0.0005; particle density: Kruskal–Wallis test: *p* = 0.0008, Dunn’s multiple comparisons test: Ctrl – 5 weeks: *p* = 0.0006 **(C)** Unbiased quantification of anti-Caspr paranode density by optical density and particle density. Statistical testing for optical density by one-way ANOVA (*p* = 0.0063) followed by Tukey’s multiple comparison test (Ctrl – 5 weeks: *p* = 0.0225, 3 weeks – 5 weeks: *p* = 0.0178) and particle density by Kruskal–Wallis test (*p* = 0.0022) followed by Dunn’s multiple comparisons test (Ctrl – 5 weeks: *p* = 0.0014), *n* = 5 mice per group. *p ≤ 0.05, ^**^p ≤ 0.01 and ^***^p ≤ 0.001.

In MS tissues, vacuolization was shown to be paralleled by structural abnormalities at Ranvier’s nodes (Luchicchi et al., 2021). To address this interesting aspect, we quantified expression levels of ankyrin G, which is required for the localization of sodium channels and the entire nodal complex (Dzhashiashvili et al., 2007). We found a strong reduction in ankyrin G immunoreactivity both in optical density and number of ankyrin G^+^ spheroidal structures during the course of cuprizone intoxication (Figure 3A upper row and Figure 3B versus optical density of PLP in Figure 1B). To verify this finding, additional stains were performed using antibodies directed against the axonal membrane protein Caspr (contactin-associated protein) which is a major component of paranodal junctions (Einheber et al., 1997). In line with the alterations of ankyrin G expression levels, the optical density and the number of Caspr^+^ spheroidal-structures (paranodes) was decreasing during the course of cuprizone-induced demyelination (Figure 3A lower row and Figure 3C).

## Discussion

Ultrastructural myelin detachments from the axon [called myelin blisters in the study of Luchicchi et al. (2021)] have been demonstrated in MS lesion rims and NAWM as well as different MS animal models. They have been demonstrated to coincide with axonal swellings, myelin protein citrullination and structural abnormalities at Ranvier’s nodes. In this study, we observed, on the histological level, remarkably similar morphological myelin alterations in the cuprizone model of metabolic oligodendrocyte injury and additionally performed SBF-SEM to track, on the ultrastructural level, these pathologies in the three-dimensional plane.

Vacuoles were evident already after 1 week of cuprizone intoxication, but more frequently found after 3 weeks of cuprizone. Vacuoles were predominantly incomplete myelin detachments with remaining contact sites toward the axon. Beyond, our results of unbiased quantification revealed that small caliber axons are more frequently affected than large caliber axons. A limitation of this quantification is that it has not been systematically investigated if demyelination (that is already persisting at the time point of 3 weeks cuprizone intoxication) affects all calibers of axons to the same extent. Predominant myelin loss of large-caliber axons at early time points has not been observed by us or others but could introduce a bias to this analysis if existent. Additionally to myelin vacuolization, axonal swellings within vacuoles were demonstrated. Of note, similar myelinated axon morphologies were found in a model of focal, lysophosphatidylcholine (LPC)-induced demyelination (El Waly et al., 2020). In that study the authors found, using intravital microscopy, axonal swellings and myelin structures reminiscent of the observed vacuoles in our study. These morphological alterations were found only days after LPC injection suggesting that vacuole formation can occur early during demyelination.

In this study, we observed a significant reduction of the expression of nodal ankyrin G and paranodal Caspr during the course of Cuprizone intoxication. This is in line with previous findings of reduction of nodal sodium channel expression (Dupree et al., 2004) and reduction of paranodal Caspr expression (Crawford et al., 2009) during cuprizone intoxication. In EAE, paranodal injury was shown to precede internodal demyelination and to coincide with early microglial activation (Howell et al., 2010; Fu et al., 2011). In MS, loss of paranodal organization was found to occur in demyelinated lesions as well as in the rim of active lesions. Additionally, paranodal disruption has been associated with early axonal pathology (Wolswijk and Balesar, 2003; Howell et al., 2006). Luchicchi et al. (2021) also reported paranodal degeneration in NAWM. Altogether, there is strong evidence that nodal and paranodal deterioration is an early event in demyelination both in MS and mouse models.

It is currently unclear, whether in MS such myelin abnormalities are induced by peripheral immune cells, can trigger or promote autoimmunity or promote the recruitment of peripheral immune cells. On the one hand, myelin vacuoles were found in the NAWM of MS patient material, where T cell densities are low (Kaddatz et al., 2021), suggesting that myelin disintegration might be an early event during MS lesion formation. On the other hand, similar abnormalities (i.e., myelinosomes) have been described in the autoimmune MS model EAE (Romanelli et al., 2016). A particularly high density of myelinosomes was present during active inflammation, suggesting that T-cell driven inflammation can trigger/support their formation. Whether indeed myelinosomes and vacuoles represent the same morphological entity and share the same underlying pathogenesis remains to be clarified in future studies. Direct comparison using the same methodological approaches would be required. Beyond, more studies are needed to understand whether such vacuoles are as well present during early MS. Nevertheless, this report adds another striking similarity between histopathological changes in progressive MS and the cuprizone model and, thus, supports the validity of this model to study the underlying mechanisms of MS disease development and progression.

## Data Availability Statement

The raw data supporting the conclusions of this article will be made available by the authors, without undue reservation.

## Ethics Statement

The animal study was reviewed and approved by the Regierung von Oberbayern (#55.2-154-2532-73-15).

## Author Contributions

MK and FS contributed to the conception and design of the study. SJ, FS, FP, CE, JK, and MF acquired and analyzed the data. MK and SJ drafted a significant portion of the manuscript and figures. All authors contributed to manuscript revision, read, and approved the submitted version.

## Conflict of Interest

The authors declare that the research was conducted in the absence of any commercial or financial relationships that could be construed as a potential conflict of interest.

## Publisher’s Note

All claims expressed in this article are solely those of the authors and do not necessarily represent those of their affiliated organizations, or those of the publisher, the editors and the reviewers. Any product that may be evaluated in this article, or claim that may be made by its manufacturer, is not guaranteed or endorsed by the publisher.

## Supplementary Material

Supplementary Figure 1 **(A)** 3D-SEM images of two representative vacuoles without a node or paranode in close proximity, asterisk: vacuole, arrowhead: axon with continuous myelin sheath. **(B)** Vacuole at partially demyelinated axon, asterisk: vacuole, black arrowheads: axon with continuous myelin sheath, white arrowheads: the same axon without myelin sheath, arrow: paranode. The same area is shown at different *z*-positions to demonstrate continuity of the axon.

Supplementary Video 1 3D-SEM image stack of axons with vacuoles; myelin is completely detached from the axon, and representative structures are pseudocolored (axon: blue, vacuole: red).

Supplementary Video 2 3D-SEM image stack of the same region as Supplementary Video 1, raw images.

Supplementary Video 3 3D-SEM image stack of axons with vacuoles; myelin is partially detached from the axon, and representative structures are pseudocolored (axon: blue, vacuole: red).

Supplementary Video 4 3D-SEM image stack of the same region as Supplementary Video 3, raw images.
