# Supplementary figures and images for: Cuprizone Intoxication Results in Myelin Vacuole Formation
Source: Front Cell Neurosci. 2022 Feb 18;16:709596. doi: 10.3389/fncel.2022.709596 (PMC8895267; doi:10.3389/fncel.2022.709596)

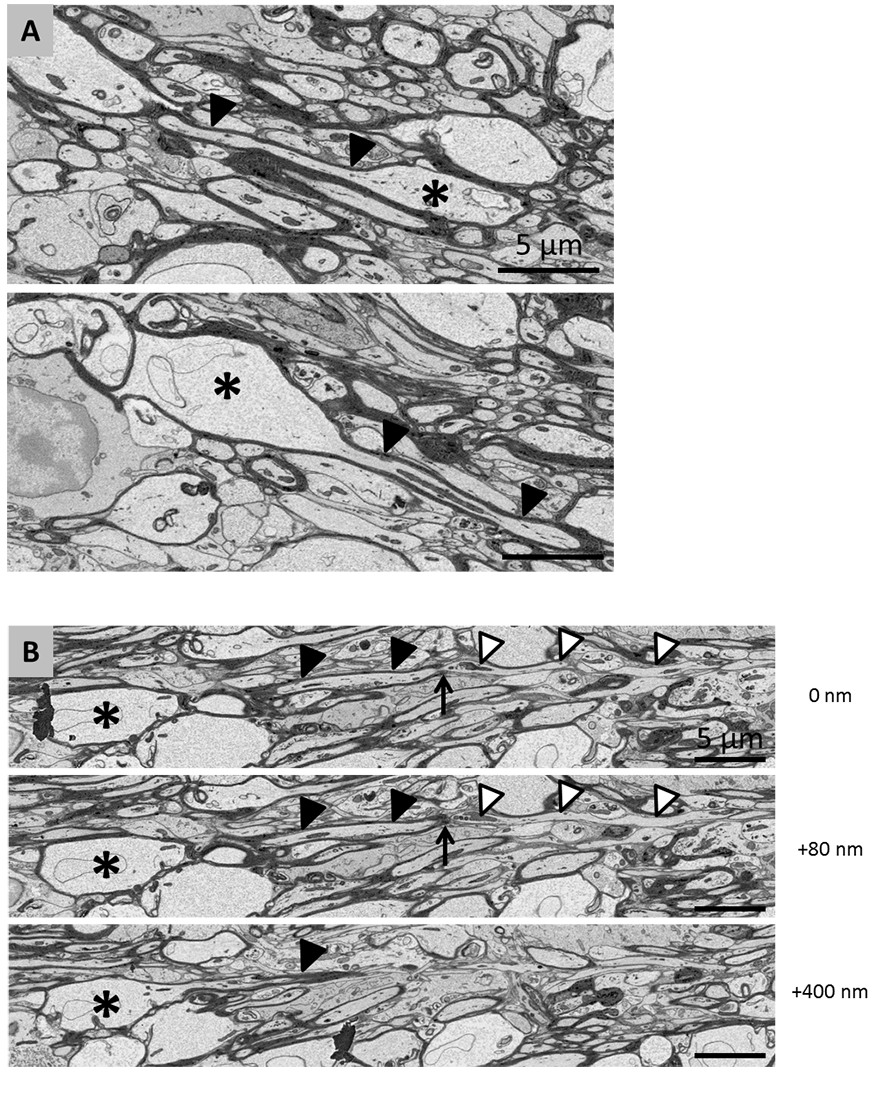

Supplement: Supplementary Figure 1 — (A) 3D-SEM images of two representative vacuoles without a node or paranode in close proximity, asterisk: vacuole, arrowhead: axon with continuous myelin sheath. (B) Vacuole at partially demyelinated axon, asterisk: vacuole, black arrowheads: axon with continuous myelin sheath, white arrowheads: the same axon without myelin sheath, arrow: paranode. The same area is shown at different z-positions to demonstrate continuity of the axon. [file Image_1.TIF]
